# Digital Assessment Approaches to Overcome Barriers to Cognitive Screening and Monitoring for Older Adults in Primary Care Settings

**DOI:** 10.1002/alz.095393

**Published:** 2025-01-09

**Authors:** Louisa I. Thompson

**Affiliations:** ^1^ Alpert Medical School of Brown University, Providence, RI USA

## Abstract

**Background:**

Routine cognitive screening for older adults in primary care could improve AD early detection and streamline referrals for treatment or clinical trials. Digital assessments, especially when self‐administered online, can overcome time barriers to cognitive screening in primary care settings and are conducive to repeat testing for disease monitoring and the evaluation of treatment outcomes. We report preliminary data on the feasibility and acceptability of three digital screening approaches for older adults completing annual follow‐up visits with a primary care provider (PCP)

**Methods:**

Cognitive screening approaches included: 1) remote online screening with the Boston Online Cognitive Assessment (BOCA) 1‐4 weeks prior to the PCP appointment, 2) self‐administered BOCA in the waiting room before or after the appointment, and 3) provider‐administered screening during the appointment using the Digital Clock and Recall (Linus Health DCR^TM^). Participants also completed a brief in‐clinic cognitive health consultation that included the Montreal Cognitive Assessment (MoCA), test feedback, and recommendations. Five PCPs aided in protocol development and participated in data collection. Potential participants were identified using EMR. Exclusion criteria: dementia or other neurological disease, score of <13 on the t‐MoCA.

**Results:**

48 older adults ages 55‐85 were screened, 34 enrolled, and 4 withdrew. The sample is 54% female, 84% White, education M = 14.8 years. Self‐administration of BOCA in waiting room was discontinued early due to space, time, and coordination challenges. Participants instead completed a second at‐home administration of the BOCA on the day of their appointment. Completion rates were 81% for advance BOCA, 62% for same‐day BOCA, and 86% for in‐clinic DCR. Higher priority health issues and not returning for the consultation visit were primary reasons for withdrawal. Most participants reported a preference for screening at home compared to in‐clinic.

**Conclusions:**

At‐home, self‐administered assessment online and in‐clinic, provider‐administered assessment on a tablet both show preliminary evidence of feasibility and acceptability for cognitive screening and monitoring use in primary care.

